# Insights from real-world data: clinical features and treatment patterns of Compound Jinqiancao Granules in patients with prostatitis

**DOI:** 10.3389/fphar.2026.1832292

**Published:** 2026-06-25

**Authors:** Yutong Ma, Wenjuan Xiong, Yanming Xie

**Affiliations:** Institute of Basic Research in Clinical Medicine, China Academy of Chinese Medical Sciences, Beijing, China

**Keywords:** Compound Jinqiancao Granules, electronic medical record (EMR), hospital information system (HIS), prostatitis, real-world study

## Abstract

**Objective:**

To analyze the clinical characteristics and combination medication patterns of Compound Jinqiancao Granules for prostatitis in a real-world setting, and to inform the rational and safe clinical use of this preparation.

**Methods:**

This study comprehensively analyzed electronic medical record (EMR) data from 1,922 patients who received Compound Jinqiancao Granules. The data were extracted from the Hospital Information System (HIS) databases of 20 tertiary hospitals nationwide. Using analytical methods such as association rule mining and complex network analysis, along with algorithms like Apriori and Louvain, we systematically investigated the clinical medication characteristics and combination medication patterns.

**Results:**

Most patients were aged 46–65 years. Admissions were most frequent in the Urology Department, with the highest admission volume occurring during the Rain Water solar term. Hospital stays typically ranged from 8 to 14 days. Prostatitis was the most common primary biomedical diagnosis. Frequently combined biomedical agents included finasteride, diuretics, hemostatics, antibiotics, and glucocorticoids. Concomitant Chinese proprietary preparations followed syndrome differentiation principles: patients with Dampness-Heat Toxin Accumulation often received Compound Kushen Injection, whereas those with Qi Stagnation and Blood Stasis commonly received Yuanhu Zhitong Dropping Pills.

**Conclusion:**

This study describes the real-world clinical use and medication patterns of Compound Jinqiancao Granules for prostatitis. Dosage, duration, and administration generally aligned with the drug labeling, and co-medication reflected routine clinical practice for prostatitis. Given the absence of a control group and adjustment for confounding factors, no causal inferences regarding efficacy or therapeutic effectiveness can be drawn. Keywords.

## 1 Introduction

Prostatitis is the most prevalent inflammatory disease of the male reproductive system, predominantly affecting young and middle-aged men. Its pathogenesis is associated with both pathogenic microorganism infections and non-infectious factors. Patients typically present with local or systemic symptoms such as pelvic pain and voiding abnormalities, and the disease is characterized by a recurrent and persistent clinical course (Xie, 2021). Prostatitis can affect men across all age groups, with a higher incidence observed in those aged 20-59 years. Globally, the prevalence of chronic prostatitis ranges from 2.2% to 9.7% (Guu et al., 2018), while the prevalence among the Chinese male population is 8.4%. Among all prostatitis cases, Type I accounts for less than 1%, Type II for 10%, and Type III for approximately 90%. Furthermore, the overall prevalence of sexual dysfunction caused by this disease is 62% (Gao et al., 2022).

In modern medicine, the most commonly used pharmacological agents for prostatitis include α-receptor blockers, antibiotics, and non-steroidal anti-inflammatory drugs. In traditional Chinese medicine (TCM), prostatitis falls under the categories of “Jing Zhuo” (seminal turbidity) (Chen, 2021) and “Lin Zheng” (stranguria) (Wu and Shi, 2021). Its pathogenesis is often attributed to irregular diet, such as excessive consumption of alcohol and rich, fatty, or sweet foods, which leads to the accumulation of Dampness-Heat. It can also result from external contraction of Dampness-Heat pathogens or unhygienic sexual activity, allowing Dampness-Heat to invade via the seminal tract and accumulate in the lower energizer. Compound Jinqiancao Granules, which clear heat, promote diuresis, and alleviate stranguria, are used for urinary tract infections caused by Dampness-Heat pouring downward. The formula is derived from a folk prescription of the Guangxi Zhuang ethnic group. It comprises Desmodii Styracifolii Herba (Guang Jinqiancao), Plantaginis Herba (Cheqiancao), Pyrrosiae Folium (Guangshijue), and Maydis Stigma (Yumixu)—all medicinal materials that are traditionally used by the Zhuang people and are abundant in the Zhuang autonomous region.

In this formula, Desmodii Styracifolii Herba (Guang Jinqiancao), sweet in taste and cool in nature, serves as the monarch drug, exerting effects of clearing heat, eliminating dampness, promoting diuresis, and alleviating stranguria. According to the Guangdong Chinese Materia Medica (Guangzhou Municipal Health Bureau Institute for Drug Control, 1963), it “calms liver fire, promotes diuresis, relieves stranguria, and clears dampness-heat.” Plantaginis Herba (Cheqiancao), sweet and cold in nature, clears heat and promotes diuresis. The Treatise on the Properties of Medicinals (Zhen, 1983) states that it “can tonify the five viscera, improve vision, promote urination, and relieve five types of stranguria.” Pyrrosiae Folium (Guangshijue), bitter, sweet, and slightly cold in nature, promotes diuresis, alleviates stranguria, and clears dampness-heat. The Shennong’s Classic of the Materia Medica (Wu et al., 2016) describes it as “primarily treating fatigue-heat pathogenic qi, urinary retention and obstruction, and promoting urination and water passage.” Maydis Stigma (Yumixu) acts by bland percolation to eliminate dampness, promoting diuresis and reducing edema. The combination of these botanical drugs achieves the synergistic effects of clearing heat, eliminating dampness, and alleviating stranguria.

Modern pharmacological studies (Zhou et al., 2011; Wu et al., 2012; Pan, 2020; Zhao, 2015; Jia et al., 2013; Jia, 2013) have demonstrated that Compound Jinqiancao Granules possess anti-inflammatory, antibacterial, diuretic, and nephroprotective effects. This preparation has been included in the Pharmacopoeia of the People’s Republic of China from the 2010 to 2020 editions and has been consistently listed in the National Reimbursement Drug List for Basic Medical Insurance, Work-Related Injury Insurance, and Maternity Insurance from 2004 to 2023.

Previous real-world studies have predominantly focused on the broad-spectrum therapeutic effects of Chinese botanical drugs in treating urinary tract infections such as urolithiasis and urethritis. However, large-sample, multi-center investigations into the clinical characteristics and medication patterns of CCPP specifically for prostatitis remain insufficient. This study, for the first time, identified a cohort of 1,922 patients definitively treated with this medication for prostatitis. Based on HIS data from 20 tertiary hospitals, we systematically analyzed clinical characteristics including age distribution, solar term correlations, and departmental disposition, as well as the synergistic patterns of integrated Chinese and biomedicine combination therapies. These findings provide descriptive real-world associations to inform the clinical positioning of this medication in prostatitis management.

This retrospective real-world study aimed to explore two exploratory hypotheses: first, that Compound Jinqiancao Granules are predominantly prescribed for prostatitis in middle-aged patients, with distinct seasonal admission patterns correlated with TCM dampness-heat pathogenesis; second, that concomitant medications follow syndrome differentiation principles and routine prostatitis management, reflecting safe and rational polypharmacy. The primary objective was to describe the clinical characteristics, medication patterns, and safety profiles of Compound Jinqiancao Granules for prostatitis; the secondary objective was to explore seasonal and syndrome-specific medication associations. As a multi-center real-world investigation, it included 1,922 patients from 20 tertiary hospitals, ensuring a large and representative sample. Association rule mining and complex network analysis were employed as standard approaches to characterize medication patterns.

## 2 Materials and methods

Botanical identification and authentication:All botanical drugs in Compound Jinqiancao Granules were taxonomically authenticated by Guangxi Wantong Pharmaceutical Co., Ltd. Voucher specimens were deposited in the herbarium of Guangxi Wantong Pharmaceutical Co., Ltd. The scientific nomenclature was validated using the Medicinal Plant Names Service (MPNS, http://mpns.kew.org/mpns-portal/) and Plants of the World Online (POWO, http://www.plantsoftheworldonline.org). The botanical drugs included in the preparation are: 1. Desmodium styracifolium (Osbeck) Merr. [Leguminosae; Desmodii Styracifolii Herba] 2. Plantago asiatica L. [ Plantaginaceae; Desmodii Styracifolii Herba] 3. Pyrrosia sheareri (Bak.) Ching [Polypodiaceae; Pyrrosiae Folium] 4. Zea mays L. [Gramineae; Stigma Maydis].

The preparation is manufactured via water extraction followed by granulation. According to the official specification, each 3 g granule is equivalent to 4.9 g of raw botanical drugs, representing a “drug–extract ratio of 4.9:3 (approximately 1.63:1)”. The final dosage form is a brown to brownish granule for oral administration. All botanical drugs are authentic, non-endangered, and comply with the requirements of the Chinese Pharmacopoeia.

### 2.1 Data source

From the massive real-world database established by the Institute of Basic Research in Clinical Medicine, China Academy of Chinese Medical Sciences (Yang et al., 2011), we extracted electronic medical record information of 1,922 patients who received Compound Jinqiancao Granules across 20 large-scale tertiary hospitals nationwide. The extracted data primarily encompassed basic patient information, diagnostic records, and medication details.

All botanical drugs used in the preparation are derived from non-endangered, commonly cultivated species as specified in the Chinese Pharmacopoeia, with no restricted or endangered species included.

Three orthogonal fingerprinting methods were applied according to ConPhyMP guidelines and pharmacopoeial requirements: High-performance liquid chromatography with diode array detection (HPLC-DAD) Ultra-performance liquid chromatography coupled with mass spectrometry (UPLC-MS) Thin-layer chromatography (TLC).

### 2.2 Study population

This was a retrospective, real-world study based on electronic medical records (EMRs) from 20 tertiary hospitals in China. A total of 1,922 male patients diagnosed with prostatitis and treated with Compound Jinqiancao Granules were enrolled.

Inclusion Criteria

1. Male patients with a primary biomedical diagnosis of prostatitis based on ICD-10 codes.
2. Patients who received Compound Jinqiancao Granules during hospitalization or outpatient treatment.
3. Patients with complete basic demographic, diagnostic, medication, and outcome data available for analysis.

Exclusion Criteria

1. Patients with a known history of allergy or hypersensitivity to any botanical drug in Compound Jinqiancao Granules.
2. Patients with severe hepatic insufficiency, severe renal insufficiency, severe heart failure, or malignant tumors that could significantly confound outcome assessment.
3. Duplicate or invalid medical records.

### 2.3 Data standardization and normalization

The collected database information underwent rigorous standardization and normalization. biomedicine diagnoses were standardized according to the coding criteria of the International Statistical Classification of Diseases and Related Health Problems, 10th Revision (ICD- 10). Traditional Chinese medicine (TCM) syndrome information was normalized by referencing the Classification and Codes of TCM Diseases and Syndromes (2021 Edition). Biomedicines were standardized using the Anatomical Therapeutic Chemical (ATC) classification system. For CCPP already assigned national reimbursement codes, standardization was performed based on their existing codes; those without assigned codes were first coded according to the national reimbursement code compilation rules prior to standardization. This process ensured the uniqueness of patient information, the linkage between different data tables, the consistency between medical orders and diagnostic names, and the validity of medication dosage units and laboratory test results, thereby establishing a comprehensive, valid, and standardized analytical dataset.

This study strictly adhered to the Real-World Study Data Management Specifications to address inherent issues of missing data and duplicate records in the HIS database, employing targeted strategies to ensure data quality. Missing data were handled by multiple imputation for key variables (age, diagnosis, medication). For patients with missing data accounting for more than 30% of key information (e.g., age, diagnosis, medication), they were excluded from the subsequent stratified analysis to ensure the reliability of results. Meanwhile, multi-center heterogeneity was effectively controlled by adopting standardized ICD-10 coding for diagnoses and unified TCM syndrome classification criteria. Outliers were identified using descriptive statistics and clinical plausibility checks, and coding errors were corrected via standardized coding and triple data verification prior to association rule mining and complex network analysis.

Additionally, strict data quality control procedures were performed, including triple manual verification, duplicate record removal, outlier screening, and missing data filtering.

### 2.4 Data analysis

Systematic analyses were conducted based on the standardized and normalized information. This is a retrospective, non-interventional, descriptive real-world study without a control group or confounding adjustment; therefore, no causal inference can be drawn. It should be noted that this retrospective study inherited inherent selection bias and information bias from electronic medical record (EMR) data, as retrospective studies are inevitably limited by the completeness and uniformity of existing clinical records. To intuitively present the bias control measures adopted in this study, the specific details are summarized in Table 1. These analyses encompassed: (1) clinical characteristic analysis, including general characteristics (age, sex, admission condition, occupation, etc.), admission characteristics, diagnoses, and medication profiles; (2) descriptive outcome analysis of clinical responses across age groups; (3) association rule mining based on the Apriori algorithm; (4) complex network analysis of combination medication data using the Louvain algorithm; and (5) safety analysis characterizing populations with abnormal liver and kidney function and identifying associated factors.

**TABLE 1:** Bias control measures.

Bias type	Control measures	Implementation details
Selection bias	Strict inclusion/exclusion criteria and duplicate record removal	Enrolled only male patients with primary prostatitis (ICD-10) who received compound jinqiancao granules; excluded those with drug allergies, severe comorbidities, duplicate or invalid medical records
Information bias	Standardized coding and triple data verification	Biomedicine diagnoses (ICD-10), TCM syndromes (2021 classification) and biomedicines (ATC) were standardized; patient data were triple-verified via ID, admission number and diagnosis timestamp to ensure documentation consistency
Missing data bias	Multiple imputation and strict exclusion criteria	Multiple imputation combined with clinical logic inference was used to supplement key missing data (age, diagnosis, medication dosage); patients with a missing rate over 30% were excluded from descriptive outcome stratification analyses to avoid confounding results
Multi-center heterogeneity bias	Unified data standardization and normalization	Data from 20 hospitals were standardized to reduce multi-center heterogeneity

This is a descriptive retrospective real-world study without a control group or statistical adjustment for confounding factors; therefore, no causal inference or treatment efficacy can be established. Solar term-based analyses were included as exploratory hypothesis-generating analyses to explore potential seasonal patterns in prostatitis presentation, reflecting traditional Chinese medicine principles, rather than to establish direct clinical utility.

### 2.5 Outcome definition

1. Complete response: Complete disappearance of clinical symptoms; normalization of objective laboratory indicators including urinary routine and prostate fluid examination.
2. Partial response: ≥50% reduction in symptom score; partial normalization of indicators.
3. No response: ≥50% improvement in clinician-assessed symptoms; partial normalization of objective laboratory indicators.

Outcomes were determined by objective indicators and physician’s evaluation, clearly distinguished in EMR records. Objective indicators included urinary routine, prostate fluid examination; subjective indicators included symptom assessment by physicians. Standardized validated questionnaires such as the National Institutes of Health Chronic Prostatitis Symptom Index (NIH-CPSI) were not used in this retrospective real-world study. Symptom assessment relied on routine clinical documentation rather than formal standardized scoring tools. All outcomes reflect descriptive clinical response based on routine EMR documentation, not standardized efficacy evaluation.

## 3 Results

### 3.1 Baseline characteristics of patients

A total of 1,922 patients were included in this study. The median age of the patients was 57 years, with the largest proportion (34.08%) aged 46–65 years. The most common hospital stay duration was 8–14 days (29.08%). The primary diagnosis was prostatitis in 36.89% of cases, followed by benign prostatic hyperplasia (6.35%) and hypertension (5.96%). The baseline demographic and clinical characteristics are summarized in Table 2.

**TABLE 2:** Baseline characteristics.

Characteristic	Value
Age (years), median (range)	57 (13–97)
Age group, n (%)	
18–45 years	460 (23.93)
46–65 years	655 (34.08)
66–85 years	462 (24.04)
≥86 years	345 (17.95)
Treatment duration (days)	
Length of hospital stay, n (%)	
8–14 days	559 (29.08)
15–28 days	549 (28.56)
Other	814 (42.34)
Top 3 primary diagnoses and common comorbidities, n (%)	
Prostatitis	2440 (36.89)
Benign prostatic hyperplasia	420 (6.35)
Hypertension	394 (5.96)

### 3.2 Admission characteristics

The majority of patients were admitted through the Urology outpatient department, consistent with the therapeutic indications. Most patients presented with general conditions, and the length of hospital stay was typically 8–14 days. Detailed admission characteristics are presented in Tables 3–6.

**TABLE 3:** Distribution of admitting departments.

Department	Frequency (n)	Percentage (%)
Urology	402	20.916
Gastroenterology	228	11.863
General surgery	155	8.065
Nephrology	108	5.619
Endocrinology	85	4.422
Hematology	77	4.006
Hepatobiliary surgery	60	3.122
Neurology	58	3.018
Cardiology	53	2.758
Oncology center	52	2.706
Traditional Chinese	43	2.237
Medicine		
Respiratory medicine	32	1.665
Internal medicine	31	1.613
Cardiovascular surgery	6	0.312
Neurosurgery	4	0.208
Others	528	27.471
Total	1922	100

**TABLE 4:** Distribution of admission routes.

Admission route	Frequency (n)	Percentage (%)
Outpatient	1632	84.912
Emergency	190	9.886
Others	100	5.203
Total	1922	100

**TABLE 5:** Distribution of admission conditions.

Admission condition	Frequency (n)	Percentage (%)
General	1141	59.365
Acute/Severe	109	5.671
Critical	12	0.624
Missing	660	34.339
Total	1922	100

**TABLE 6:** Distribution of length of hospital stay.

Length of stay (days)	Frequency (n)	Percentage (%)
≤3	97	5.047
4-7	314	16.337
8- 14	559	29.084
15-28	549	28.564
≥28	392	20.395
Missing	11	0.572
Total	1922	100

### 3.3 Clinical diagnostic characteristics

The most frequent biomedical diagnosis was prostatitis, with 2,440 diagnostic episodes (36.891% of all recorded diagnoses), followed by benign prostatic hyperplasia (BPH, 420 episodes, 6.350%) and hypertension (394 episodes, 5.957%).

TCM syndrome data were extracted from standardized electronic medical records, and syndrome differentiation was determined by attending physicians in accordance with national TCM syndrome classification criteria. In terms of Traditional Chinese Medicine (TCM) syndrome differentiation, the most common pattern was Dampness-Heat Accumulation, observed in 91 cases (9.267%). This was followed by Dual Deficiency of Yin and Yang in 59 cases (6.008%), Kidney Deficiency in 52 cases (5.295%), Spleen-Kidney Deficiency in 36 cases (3.666%), and patterns of Phlegm-Stasis (26 cases, 2.648%) and Blood Stasis (26 cases, 2.648%). The remaining patients were classified as other undefined TCM syndrome types not included in the above common patterns.

### 3.4 Clinical medication characteristics

Among the 1,922 patients treated with Compound Jinqiancao Granules for prostatitis, dosage information was recorded for 824 cases. Regarding dosage specifications, the most frequently administered dose was 3 g per administration (equivalent to one sachet), accounting for 44.053% of documented cases, followed by 6 g per administration (equivalent to two sachets), representing 32.039% of documented cases. Cumulatively, dosages conforming to pharmaceutical labeling instructions accounted for 76.092% of documented cases, indicating the existence of off-label dosing practices in real-world clinical settings and underscoring the necessity for standardized medication protocols. Regarding administration methods, oral administration of Compound Jinqiancao Granules across all specifications predominated, comprising 93.689% of documented cases, which aligns with labeled instructions. Regarding treatment duration, given the inherent missing data and confounding variables in real-world research, hospitalization duration was employed as a reference metric for medication course. Analysis revealed that patients receiving Compound Jinqiancao Granules for prostatitis predominantly experienced hospitalization durations of 8-14 days (559 cases, representing 29.084% of the total cohort), followed by 15-28 days (549 cases, accounting for 28.564% of the total cohort). These findings correspond to the therapeutic patterns characteristic of prostatitis management, with the relatively extended hospitalization periods attributable to the high prevalence of comorbid internal medical conditions within this patient population.

### 3.5 Concomitant medications and pharmacological actions

In patients receiving Compound Jinqiancao Granules for prostatitis, the most frequently co-prescribed biomedicines were potassium chloride and furosemide. Prostatitis commonly induces urinary dysfunction, frequently necessitating the administration of diuretic agents. Furosemide, as a potassium-depleting diuretic, requires concomitant potassium supplementation to prevent hypokalemia. Following these, dexamethasone and levofloxacin were frequently utilized, representing standard pharmacotherapeutic agents for genitourinary tract infections, employed to achieve anti-inflammatory and anti-infective effects while alleviating urethral pressure. These concomitant medication patterns correspond to the pathophysiological progression of the disease and its associated therapeutic requirements.

Regarding concomitant Traditional Chinese Medicine (TCM) preparations, the most frequently co-administered were Chaihu Injection (Bupleurum Injection), Shuxuetong Injection (Leech and Earthworm Injection), Xiyanping Injection (Andrographolide Sulfonate Injection), and Montmorillonite Powder. These agents were employed to address bacterial-induced prostatitis, manage associated low-grade febrile responses, or provide antidiarrheal therapy for patients presenting with concomitant diarrhea.

### 3.6 Association rule analysis of concomitant medications

**FIGURE 1:**
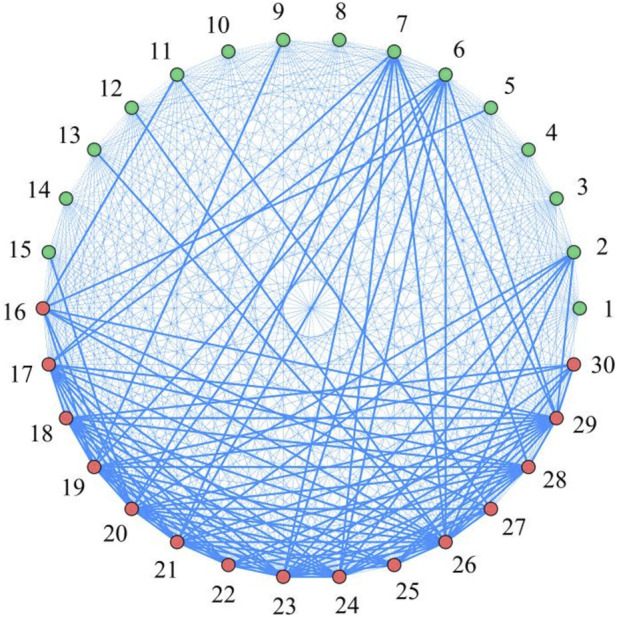
Association rule diagram of the top 15 co-prescribed biomedicines and Traditional Chinese Medicines.

**FIGURE 2:**
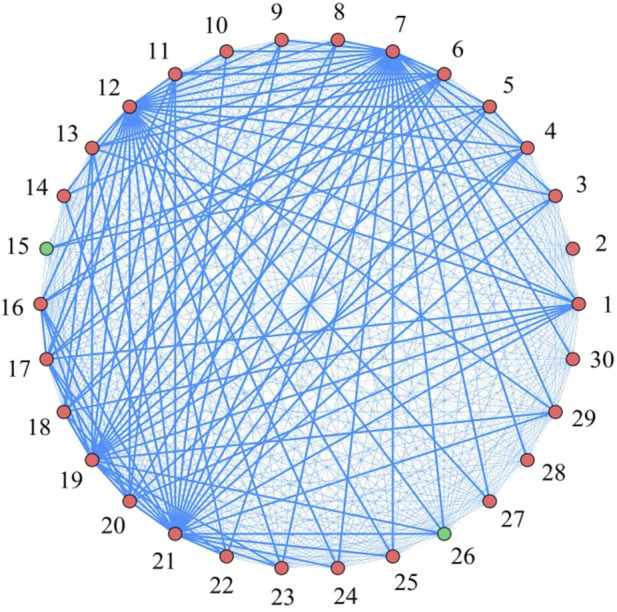
Association rule diagram of the top 30 co-prescribed medication categories.

From the two-item frequent sets identified through Apriori algorithm analysis, after filtering out biomedicine combinations, TCM-related pairs were retained. The top 15 concomitant biomedicine and TCM combinations were extracted to generate association rule diagrams (Figure 1). Similarly, the top 30 concomitant medication categories were extracted for visualization (Figure 2). In Figure 1, varying line styles represent co-prescription frequencies: bold solid lines indicate high-frequency associations (≥1.83%), thin solid lines represent medium-frequency associations (0.37%-1.83%), and dashed lines denote low-frequency associations (≤0.37%), providing an intuitive visualization of clinically significant synergistic relationships between biomedicine and Traditional Chinese Medicines.

The analysis revealed frequent co-prescription patterns involving Aspirin, Amlodipine, Omeprazole, Dexamethasone, Sennosides, Furosemide, Metoclopramide, Hyoscyamine, Potassium Chloride, Magnesium Aspartate, Pantoprazole, Sodium Bicarbonate, Cefazolin, Vitamin C, and Levofloxacin, in conjunction with Chaihu Injection (Bupleurum Injection), Danhong Injection (Salvia Miltiorrhiza and Safflower Injection), Ganmao Qingre Granules (Common Cold Relieving Granules), Kangfuxin Liquid (*Periplaneta Americana* Extract), Montmorillonite Powder, Relinqing Granules (Heat Cleansing and Stranguria Relieving Granules), Shuxuetong Injection (Leech and Earthworm Injection), Simotang Oral Liquid (Four Milled Decoction Oral Liquid), and Yunnan Baiyao (Powder/Capsules).

Figure 2 employs medication categories as analytical units, with visual line differentiation representing co-prescription frequencies across therapeutic classes: bold solid lines indicate high-frequency associations (≥6.76%), thin solid lines represent medium-frequency associations (2.99%-6.76%), and dashed lines denote low-frequency associations (≤2.99%). The visualization reveals prevalent co-prescription patterns among the following therapeutic categories: β-lactam antibacterials, antipsychotics, aminoglycoside antibacterials, phenylpiperidine derivatives, non-steroidal anti-inflammatory and antirheumatic agents, calcium channel blockers, hepatotherapeutic agents, antihemorrhagics, anti-infectives, antacids antithrombotic agents, mineral supplements, quinolone antibacterials, diuretics, immunostimulants, corticosteroids, other antibacterials, expectorants, osmotic laxatives, vitamins, gastrointestinal prokinetics, cardiac therapeutics, analgesics, antiemetics, constipation treatments, functional gastrointestinal disorder medications, peptic ulcer disease treatments, stasis-transforming and vessel-dredging agents, and heat-clearing and detoxifying preparations.

### 3.7 Patient cross-analysis

The quantitative relationship between clinical medication and treatment outcomes in patients receiving Compound Jinqiancao Granules for prostatitis is presented in Tables 7, 8. Among the 1,922 patients, a total of 6,614 treatment outcome records were documented for primary diagnoses, including 1,197 cases (18.098%) of complete response and 4,113 cases (62.186%) of partial response. The overall response rate was 80.284%, calculated as: Overall response rate (%) = (Number of complete responses + Number of partial responses)/Total number of outcome records × 100%. Patient ages were predominantly distributed between 18-85 years, with an overall clinical response rate of 95.435%, 96.641%, and 96.320% observed in the 18-45, 46-65, and 66-85 age groups, respectively. Patients aged 46–65 years accounted for the highest proportion (34.079%) and showed the best clinical response.

**TABLE 7:** Distribution of treatment outcomes.

Treatment outcomes	Frequency (n)	Percentage (%)
Complete response	1197	18.098
Partial response	4113	62.186
No response	36	0.544
Deceased	18	0.272
Transferred	1250	18.899
Total	6614	100

**TABLE 8:** Distribution of complete and partial response rates by age group.

Age (years)	Frequency (n)	Percentage (%)	Number of clinical response cases in age group	Percentage in age group (%)
<18	5	0.260	/	/
18∼45	460	23.933	439	95.435
46∼65	655	34.079	632	96.641
66∼85	462	24.037	445	96.320
≥86	36	1.873	/	/
Missing	304	15.817	/	/
Total	1922	100	/	/

### 3.8 Correlation between solar terms and admission frequency

Regarding admission distribution across solar terms, the highest number of admissions occurred during the Rain Water solar term (111 patients, 5.78%), followed by Minor Cold (100 patients, 5.20%), Grain in Ear, and Minor Heat (each 95 patients, 4.94%). Detailed data are presented in Table 9; Supplementary Figure S1. Medically, the peak during Rain Water aligns with the TCM dampness-heat pathogenesis of prostatitis. Elevated humidity and temperature fluctuations during this period may exacerbate lower urinary tract symptoms, showing a seasonal correlation consistent with TCM dampness-heat theory. Its clinical implications remain unclear and exploratory, requiring further investigation.

**TABLE 9:** Distribution of admissions by solar terms.

Solar term	Frequency (n)	Percentage (%)
Beginning of spring (Li Chun)	66	3.434
Rain water (Yu Shui)	111	5.775
Awakening of insects (jJing Zhe)	79	4.11
Spring equinox (Chun en)	82	4.266
Pure brightness (Qing Ming)	90	4.683
Grain rain (Gu Yu)	93	4.839
Beginning of summer (Li Xia)	88	4.579
Grain full (xiao Man)	86	4.475
Grain in Ear (Mang Zhong)	95	4.943
Summer solstice (Xia Zhi)	83	4.318
Minor heat (Xiao Shu)	95	4.943
Major heat (Da Shu)	70	3.642
Beginning of autumn (Li Qiu)	74	3.85
End of heat (Chu Shu)	88	4.579
White dew (Bai Lu)	70	3.642
Autumn Equinox (Qiu Fen)	53	2.758
Cold dew (Han Lu)	65	3.382
Frost descent (Shuang Jiang)	73	3.798
Beginning of winter (Li Dong)	75	3.902
Minor snow (Xiao Xue)	72	3.746
Major snow (Da Xue)	78	4.058
Winter solstice (Dong Zhi)	79	4.11
Minor cold (Xiao Han)	100	5.203
Major cold (Da Han)	57	2.966
Total	1922	100

## 4 Discussion

This study is a correlational analysis only, not a causal investigation. Observed patterns are descriptive, and their medical implications are unclear and exploratory at present.

### 4.1 Baseline characteristics of treated patients

Regarding age distribution, among the 1,922 patients treated with Compound Jinqiancao Granules for prostatitis, 460 patients were aged 18-45 years, of whom 439 achieved complete or partial response, accounting for 95.44% of this age group. Epidemiological surveys indicate that prostatitis predominantly affects individuals aged 20-59 years, thus the 18-45 years age group aligns with the general epidemiological pattern of prostatitis. Regarding clinical diagnoses, prostatitis constituted the highest proportion (36.89%), followed by benign prostatic hyperplasia and other conditions, consistent with the clinical reality that patients with prostatitis often present with concomitant internal medical disorders. Regarding administration routes, oral administration was predominant, accounting for 93.69% of cases. Concerning dosage, 76.09% of patients received doses compliant with the drug instructions, primarily 3 g or 6 g per administration. Length of hospital stay was predominantly concentrated in the 8-14 days and 15-28 days ranges, accounting for 29.08% and 28.56% respectively, consistent with the protracted and recurrent nature of prostatitis requiring adequate treatment courses. Patients were primarily admitted through the Urology outpatient department (20.92%), highly consistent with the therapeutic indications of Compound Jinqiancao Granules for urinary tract infections, with admission conditions predominantly classified as general (59.37%), reflecting routine clinical practice scenarios. As an exploratory analysis, solar term distribution showed the highest number of admissions during the Rain Water (5.78%) and Minor Cold (5.20%) solar terms. These exploratory findings suggest potential seasonal patterns; however, this peak may be affected by post-holiday healthcare utilization patterns in China, as the Rain Water term often occurs shortly after the Spring Festival. Such socioeconomic confounders may influence the observed seasonality independent of disease biology.

From the perspective of the TCM theory of “correspondence between heaven and human,” the Rain Water solar term is characterized by climatic features of “predominance of Dampness pathogen and alternating cold and warm conditions.” During this period, natural water vapor rises, and the Dampness pathogen becomes prevalent, exhibiting properties of “heaviness, turbidity, stickiness, and downward tendency.” This pathogen readily invades the lower energizer of the human body, combining with pre-existing internal Dampness-Heat to form “Dampness-Heat intermingling,” which obstructs qi movement and impedes blood circulation, thereby exacerbating symptoms such as frequent urination, urinary urgency, and perineal distension and fullness. Concurrently, the Dampness pathogen tends to obstruct qi movement in the lower energizer, inducing “Qi Stagnation and Blood Stasis,” which intensifies pelvic pain and other symptoms. Moreover, the unstable cold-warm conditions characteristic of this solar term readily consume the gradually declining kidney qi of middle-aged and elderly patients, diminishing the body’s defensive capacity and disrupting water-dampness transformation and transportation, further aggravating voiding abnormalities. These pathogenic mechanisms ultimately prompt patients to seek medical consultation, suggesting that the interplay of external climatic factors and internal pathogenesis may be associated with seasonal presentation of prostatitis.

### 4.2 Combination medication patterns

#### 4.2.1 Biomedicines

Pharmacological studies have demonstrated that Compound Jinqiancao Granules possess anti-inflammatory, antibacterial, diuretic, and nephroprotective effects (Zhao, 2015; Jia et al., 2013; Jia, 2013; Yang et al., 2011; Wu et al., 2024). In clinical practice for prostatitis treatment, the selection of concomitant biomedicines shows close alignment with the disease’s pathological mechanisms (such as infection control, improvement of voiding symptoms, and prevention of complications) and the need for synergistic therapeutic effects. Analysis of clinical medication data reveals that the most frequently combined biomedicines with Compound Jinqiancao Granules for prostatitis exhibit clear disease-specific relevance: For the common voiding dysfunction symptoms in prostatitis patients, potassium-wasting diuretics such as furosemide are frequently combined to promote urine output and alleviate urethral obstruction sensation. However, given furosemide’s propensity to induce hypokalemia, potassium chloride supplements are often concurrently administered to maintain electrolyte balance and prevent adverse reactions. For patients with bacterial prostatitis or concurrent urinary tract infections, antimicrobials such as levofloxacin (fluoroquinolone) and cefazolin (β-lactam) are commonly combined. Levofloxacin, due to its potent antimicrobial activity against common urogenital pathogens (e.g., *Escherichia coli*, *Staphylococcus* species) and favorable tissue penetration, emerges as a high-frequency concomitant medication, while cefazolin is typically employed in scenarios requiring coverage against Gram-positive bacterial infections. For patients presenting with concomitant pelvic pain, gastrointestinal disturbances, or underlying diseases, clinical practice additionally incorporates non-steroidal anti-inflammatory drugs for pain relief, acid-suppressing agents such as omeprazole for gastrointestinal discomfort, and amlodipine for managing comorbid hypertension. These concomitant medications are commonly used in clinical practice to address pain, comorbidities, and related symptoms, consistent with the real-world clinical needs of multi-disease comanagement and symptomatic intervention for prostatitis.

#### 4.2.2 Chinese medications

Analysis of clinical medication data reveals that the categories and specific Chinese medicines combined with Compound Jinqiancao Granules for prostatitis treatment exhibit clear syndrome-specific relevance: For patients with pronounced Dampness-Heat Toxin Accumulation syndrome, Compound Kushen Injection is frequently combined. This preparation possesses effects of clearing heat, resolving toxins, cooling blood, and removing blood stasis. When used together with Compound Jinqiancao Granules, these combinations are frequently employed in clinical practice and warrant further prospective validation for patients with marked dampness-heat toxin symptoms such as urethral burning, dark yellow urine, and local redness and swelling. For patients with Qi Stagnation and Blood Stasis syndrome, Yuanhu Zhitong Dropping Pills are commonly combined clinically, which regulate qi, activate blood, unblock collaterals, and alleviate pain. For patients presenting with concomitant low-grade fever or externally-contracted symptoms, Chaihu (Bupleurum) Injection is frequently combined; this medication relieves exterior syndromes, reduces fever, soothes the liver, and regulates qi, simultaneously alleviating low-grade fever discomfort and regulating qi movement in the lower energizer. Additionally, for patients with Dampness-Heat complicated by Kidney Deficiency syndrome, Chinese medicine preparations with kidney-tonifying actions are often used as adjunctive therapy. Such combinations aim to nourish kidney qi while clearing heat and eliminating dampness, and represent commonly applied clinical approaches in real-world practice.

Overall, the combination of Compound Jinqiancao Granules with other Chinese medicines strictly adheres to the TCM principle of treatment based on syndrome differentiation. By selecting appropriate medications for different syndrome types, this approach not only reinforces the treatment of the core pathogenesis of prostatitis—“Dampness-Heat pouring downward”—but also addresses concomitant syndromes and protects healthy qi, aligning with the clinical requirements of complex syndrome manifestations in real-world prostatitis treatment.

### 4.3 Medication usage

The clinical dosage, administration methods, and treatment course of Compound Jinqiancao Granules for prostatitis generally conform to the drug instructions, demonstrating favorable medication safety and appropriateness. Regarding administration routes, oral administration was predominant, accounting for 93.69% of cases, consistent with the instructions. Concerning dosage, 76.09% of patients followed the recommended dosage in the instructions, primarily receiving 3 g (one packet) or 6 g (two packets) per administration. Although a small number of cases deviated from the labeled dosage, overall prescribing practices showed high standardization, laying a foundation for clinical response and medication safety. Regarding treatment course, based on the length of hospital stay (8-14 days: 29.08%; 15-28 days: 28.56%), and considering the protracted nature of prostatitis requiring adequate treatment duration, clinical medication courses generally aligned with disease treatment cycles. Regarding treatment outcomes, among the 1,922 patients, complete and partial response cases accounted for 80.28%, with the 46-65 years age group exhibiting the highest complete and partial response rate (96.64%). These clinical response results are descriptive in nature and do not confirm treatment efficacy. However, the direct relationship between dosage, treatment course, and clinical outcomes remains unclear, warranting further investigation. Additionally, the small number of off-label prescribing instances observed in clinical practice highlight the need for enhanced medication standardization management, optimizing dosing regimens based on individual patient characteristics (such as age, comorbidities, and syndrome types) to better balance clinical response and safety, thereby ensuring the full realization of the clinical value of Compound Jinqiancao Granules in prostatitis treatment.

### 4.4 Strengths and limitations

This study analyzed 1,922 prostatitis patients treated with Compound Jinqiancao Granules using real-world HIS data from 20 tertiary hospitals. We described demographic and medication patterns, evaluated clinical response, explored optimal combinations via network analysis, and assessed safety, providing evidence for clinical application.

Safety evaluation revealed no Compound Jinqiancao Granules–related adverse events or abnormal laboratory tests were observed. No significant interactions with antibiotics or α-blockers were reported, supporting a favorable safety profile.

As a retrospective study utilizing data from the HIS databases of 20 tertiary hospitals, this investigation offers the advantages of a large sample size and high real-world authenticity. However, this retrospective study inherited inherent selection bias and information bias: selection bias may arise from the non-random inclusion of patients (only those with complete EMR records were enrolled), and information bias may result from inconsistent documentation standards of clinical symptoms and medication details among different hospitals. Nevertheless, as the data originate from clinical medical records, they are inherently subject to limitations including missing data, duplicate entries, and confounding factors. Concomitant medications commonly used for prostatitis, such as antibiotics, corticosteroids, and diuretics, were frequent in this cohort, and their potential interactions with Compound Jinqiancao Granules were not fully evaluated, which may affect outcome interpretation.

Regarding treatment duration and medication adherence, due to incomplete documentation of medication courses in real-world data, this study used length of hospital stay as a proxy for treatment course analysis. However, some patients may have continued medication after discharge or discontinued treatment prematurely, potentially introducing bias in treatment course assessment.

Concerning baseline information, data on lifestyle factors (such as alcohol consumption and prolonged sitting) and medical history were frequently missing. These factors may influence the onset and prognosis of prostatitis, potentially contributing to baseline differences between subgroups.

Regarding diagnostic standardization, although biomedicine diagnoses were standardized using ICD- 10 coding, TCM syndrome differentiation relied on clinical physicians’ judgments. Variations in diagnostic criteria among different physicians may have introduced bias in syndrome-related analytical results. Second, the evaluation of clinical response was based on routine clinical documentation in EMRs rather than standardized questionnaires such as NIH-CPSI, which is an inherent limitation of retrospective real-world studies.

Concerning the stratified analysis, due to the extremely low number of patients receiving Compound Jinqiancao Granules as monotherapy and incomplete documentation of specific concomitant medications, we could not perform subgroup comparisons by treatment regimen. As a retrospective study without a control group, causal conclusions cannot be established. All outcome assessments were based on routine clinical documentation in electronic medical records rather than standardized tools. Future randomized controlled trials (RCTs) or prospective cohort studies are warranted to validate these real-world observations.

Most importantly, this study is a retrospective, non-interventional, descriptive real-world analysis without a comparator group or adjustment for confounding variables. Accordingly, it cannot establish causal relationships or validate the therapeutic effectiveness of Compound Jinqiancao Granules. All clinical response results are purely descriptive and should not be interpreted as evidence of treatment efficacy.

### 4.5 Comparison with similar studies and complementary value of this research

Previous real-world studies on Compound Jinqiancao Granules have primarily focused on its clinical response and medication patterns in treating urinary tract stones and urethritis (Zhou et al., 2011; Jia et al., 2013), with a limited number of studies mentioning its secondary indication for “prostatitis complicated by urinary tract infection.” No dedicated analysis specifically targeting prostatitis has been conducted. For instance, Zhou et al. (Zhou et al., 2011) investigated the diuretic and antispasmodic effects of Compound Jinqiancao Granules on kidney stones, without addressing age distribution, syndrome-specific relevance, or combination medication characteristics in prostatitis patients. Wu et al. (2024) validated the preventive and therapeutic effects of this medication on chronic bacterial prostatitis through animal experiments, but their findings lacked support from large-sample clinical data.

This study, for the first time, establishes “Compound Jinqiancao Granules in the treatment of prostatitis” as the sole core focus. Based on 1,922 multicenter real-world cases, it provides descriptive clinical data that complement existing knowledge regarding this medication in a specific disease context, including population characteristics (46-65 years as the core beneficiary population), solar term correlations (highest admission rate during Rain Water), and integrated Chinese-biomedicine combination patterns (e.g., Compound Kushen Injection for Dampness-Heat Toxin Accumulation syndrome; synergistic use of potassium-wasting diuretics with potassium supplements). These findings address gaps in previous research of this nature, rendering the clinical evidence chain for Compound Jinqiancao Granules more comprehensive.

Current real-world studies on Chinese medicines for prostatitis have predominantly focused on preparations such as Qianliexin Capsules and Longjin Tonglin Capsules, with varying research emphases. Some studies have concentrated on clinical response validation of single CCPP. For instance, Wang et al. (2024) evaluated the overall response rate, safety, and cost-clinical response of Qianliexin Capsules in treating chronic prostatitis, but their study lacked in-depth analysis of combination medication patterns. Zhang et al. (2021) merely listed the types of biomedicines combined with Shumitong Capsules for treating chronic prostatitis with Dampness-Heat Stagnation syndrome, without conducting a thorough analysis of their correlation with TCM syndromes.

Compared to similar studies, this research offers several incremental contributions. First, it elucidates the high degree of specific relevance between Compound Jinqiancao Granules and the core pathogenesis of prostatitis—“Dampness-Heat pouring downward”—and systematically delineates differentiated medication regimens based on syndrome differentiation, including the combination of Compound Kushen Injection for Dampness-Heat Toxin Accumulation syndrome and Yuanhu Zhitong Dropping Pills for Qi Stagnation and Blood Stasis syndrome. Compared to the generalized medication analyses in similar studies, this approach more effectively highlights the clinical guiding value of TCM syndrome differentiation and treatment.

Second, this study innovatively incorporates analytical dimensions such as solar terms and age stratification, validating the association between disease onset and solar terms characterized by predominant dampness and cold (Rain Water and Minor Cold), as well as demonstrating an optimal complete and partial response rate of 96.64% in the core population aged 46-65 years. These findings provide modern clinical data supporting the TCM theory of “correspondence between heaven and human,” with such correlation analyses being rarely addressed in similar studies.

Furthermore, based on Apriori and Louvain algorithms, this study constructed multi-level combination medication networks encompassing “biomedicine-biomedicine,” “Chinese medicine-biomedicine,” and “drug category-category” relationships. These networks clearly illustrate clinical medication logic, including the electrolyte balance synergy between furosemide and potassium chloride and the antimicrobial spectrum complementarity between levofloxacin and cefazolin. Compared to the single-dimensional medication statistics in similar studies, this approach more accurately reflects the clinical reality of diagnosis and treatment in real-world prostatitis management.

## Data Availability

The dataset used in this study is restricted by the following terms: De-identification & Privacy: All personally identifiable information (PII) has been irreversibly removed. The data is anonymized and cannot be used to identify individual patients, complying with GDPR, HIPAA, and relevant national health regulations. Access & Sharing Controls: Access to the source data is limited to authorized researchers only. Unauthorized redistribution, commercial use, or disclosure to third parties is strictly prohibited. A Data Use Agreement (DUA) is required for data sharing between institutions. Purpose Limitation: The data is restricted to the research scope of this study (real-world analysis of Compound Jinqiancao Granules for prostatitis). Repurposing for other clinical or non-research purposes is not permitted without prior ethical re-approval. Governance: The data is managed in accordance with institutional IRB guidelines and healthcare data security policies, adhering to the principle of “minimum necessary” access. Requests to access the datasets should be directed to YM, 17805989725@163.com.
