# Supplementary figures and images for: Insights from real-world data: clinical features and treatment patterns of Compound Jinqiancao Granules in patients with prostatitis
Source: Front Pharmacol. 2026 Jun 25;17:1832292. doi: 10.3389/fphar.2026.1832292 (PMC13345932; doi:10.3389/fphar.2026.1832292)

Supplementary Figure 1 Radar chart of admission proportions across the 24 solar terms

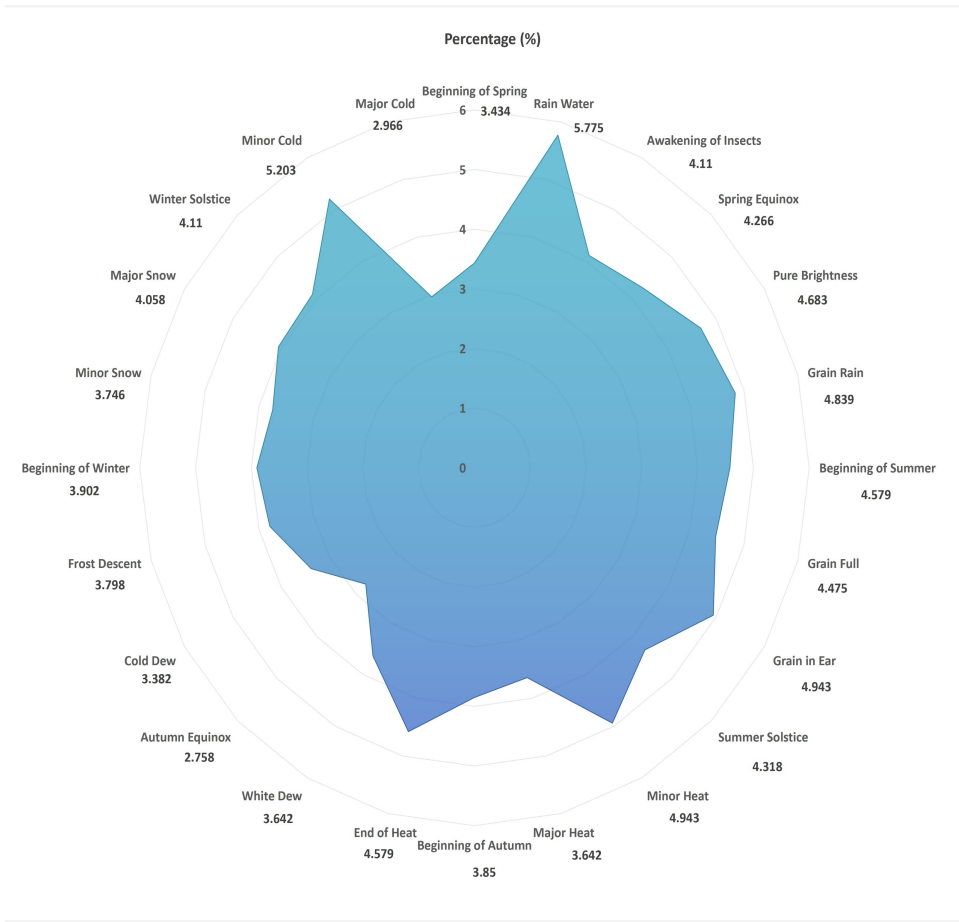

Supplement: Supplementary file 1 [file Supplementaryfile1.pdf]
